# Inherited Cancer Genetic Epidemiology to Improve Precision Medicine

**DOI:** 10.3390/jcm11030879

**Published:** 2022-02-08

**Authors:** Pål Møller, Dafydd Gareth Evans

**Affiliations:** 1 Department of Tumor Biology, Institute of Cancer Research, The Norwegian Radium Hospital, 0379 Oslo, Norway; 2 Manchester Centre for Genomic Medicine, Division of Evolution and Genomic Sciences, University of Manchester, Manchester University NHS Foundation Trust, Manchester M13 9WL, UK; Gareth.Evans@mft.nhs.uk

Epidemiology is the study of the distribution and determinants of health-related states or events in specified populations, and the application of this study to the control of health problems [1]. The current Special Issue focuses on the application of this science to inherited gastro-intestinal tumors.

Variants of genes causing/associated with cancer are denoted as pathogenic variants. When compatible with being live-born, such variants may be inherited and can cause inherited cancers to occur. In addition, variants may arise with acquired mitotic driver mutations and may cause sporadic cancers. Underlying the papers in this Special Issue are the systematic collaborative efforts that produced the international databases of recognized pathogenic variants, such as the InSiGHT variant database (https://www.insight-group.org/variants/databases/ accessed 1 February 2022), BRCA-Exchange (https://brcaexchange.org/variants accessed 1 February 2022) and ClinVar (https://www.ncbi.nlm.nih.gov/clinvar/ accessed 1 February 2022). The aims of these data resources are to summarize all of the available knowledge on which genetic variants are causing inherited cancer and to make the knowledge available to all through open access.

From the initial phase of identifying genetic variants that cause cancer, the focus is now on specifying their penetrance (cumulative incidence of cancer) and expressivities (which cancers), and how environmental factors possibly may modify these. Finding how to implement this knowledge, however, requires utilizing technical abilities at any given time in any given place as well as overall resources and local socio-ethical standards. The implementation of knowledge into health care may correspondingly differ between and within societies and over time, meaning that clinical guidelines need constant updating. Both providing more knowledge and the implementation of such is a never-ending process.

To this end, the current Special Issue includes seven papers on the determinants of factors associated with cancer, how to identify them, how to prevent and/or cure these cancers, and how to delineate inherited predispositions from sporadic tumors: the former will imply both high risk for metachronous cancers in the carriers themselves and high risk for cancers in their relatives, while the latter will not.

Five of the papers focus on five different aspects of the most frequent inherited cancer syndrome, Lynch syndrome, caused by inherited malfunctioning mismatch repair genes: Lena Bohaumilitzky et al. give an overview of the biology of the tumors and their similarities with and differences from sporadic mutations in the same genes. Karin Álvarez et al. report the tumor spectrum in Chilean families with Lynch syndrome. Neil A J Ryan et al. discuss how to identify Lynch syndrome in patients with endometrial cancer. Tristan M Snowsill et al. report the cost-effectiveness of doing so. Mev Dominguez-Valentin et al. report an international survey on risk-reducing surgery to prevent gynecological cancer in female carriers of pathogenic mismatch repair genetic variants. These five reports all add new information which will be included when the current international consensus on clinical guidelines [2,3] is updated. Mariona Terradas et al. give an update of the pathogenic variants of other genes causing hereditary non-polyposis colorectal cancer. Anja Wagner et al. give, on behalf of the European Hereditary Tumor Group (www.ehtg.org accessed 1 February 2022), updated clinical guidelines for the management of the inherited Peutz–Jeghers cancer syndrome caused by pathogenic variants of the *STK11/LKB1* gene. In this way, the current issue is part of a continuous process compliant with and endorsed by the European Hereditary Tumor Group (www.ehtg.org accessed 1 February 2022). The overall aim is to provide evidence-based individual precision medicine to prevent, detect early and cure inherited cancers.
